# Individual and community-level factors associated with modern contraceptive use among adolescent girls and young women in Ethiopia: a multilevel analysis of 2016 Ethiopia demographic and health survey

**DOI:** 10.1186/s13690-021-00736-8

**Published:** 2021-11-22

**Authors:** Samuel Hailegebreal, Binyam Tariku Seboka, Mohammedjud Hassen Ahmed, Gizaw Hailiye Teferi, Zegaye Regasa, Beemnet Tekabe, Sewunet Sako, Girma Gilano

**Affiliations:** 1 Department of Health Informatics, College of Medicine and Health Science, School of Public Health, Arba Minch University, Arba Minch, Ethiopia; 2 Department of Health Informatics, College of Medicine, and Health Science, Dilla University, Dilla, Ethiopia; 3 Department of Health Informatics, Institute of Public Health, Mettu University, Mettu, Ethiopia; 4 Department of Health Informatics, Debre-Markos University, Debre Markos, Ethiopia

**Keywords:** Adolescent, Young, Contraceptive, Multilevel analysis, EDHS, Ethiopia

## Abstract

**Background:**

The importance of contraception use is immense for young girls of age 15–24 years. In literatures, there were significant attempts made to study factors associated with adolescent and young women contraception use in Africa. Despite the resulting interventions followed those studies, the contraception uses among youth population in Africa remained below average. Thus, this study is aimed to assess individual and community-level factors associated with contraceptive use in Ethiopian context to support further interventions.

**Methods:**

Our analysis was based on the secondary data from Ethiopia Demography and Health Survey (EDHS) 2016. Adolescent girls and young women (AGYW) aged 15–24 years were the target population. Means, standard deviations, and proportions were used to describe the study population. To control for the variations due to the differences between clusters, a series of multilevel logistic regression modeling steps were followed and determinants of contraceptive use were outplayed. All variables with bivariate *p*-value < 0.25 were included in the models and *p*-value < 0.05 was used to declare associations.

**Results:**

The prevalence of modern contraceptive use among AGYW in Ethiopia was 34.89% [95% CI, 0.32, 0.36]. Married adolescents were 2.01 times [AOR = 2.01, 95% CI = 1.39,3.16], having work was 1.36 times [AOR = 1.36, 95% CI = 1.06,1.71], living in urban areas was 1.61 times [AOR = 1.61, 95% CI = 1.16,2.45], being in middle wealth status was 1.9 times [AOR = 1.90, 95% CI = 1.32,2.65], being in rich wealth quintile was 1.99 time [AOR = 1.99, 95% CI = 1.35,2.68], and having TV exposure was 1.61 times [AOR = 1.6, 95% CI = 1.17,2.20] more likely associated with modern contraceptive uses.

**Conclusion:**

The use of modern contraception among AGYW in the country remained appealing and factors like region, residence, marital status, wealth index, religion, working status, parity, husband desire children, ever aborted AGYW, and the television exposures were attributed for the poor improvements. Therefore, the enhancements that consult those factors remained remarkable in improving contraception use, while further increasing in educational engagement, access to health services, and economic empowerment of the AGYW might be the good advantages for the improvements.

## Background

Adolescents and young women (AGYW) remained the important target population for reproductive health and sexual intercourse related health issues [1]. The onset of sexual activity in AGYW is natural. With this natural onset, the knowledge of AGYW regarding contraceptive methods and the access to the important reproductive services are very imperative [2]. Educating AGYW about maternal health, reducing child mortality, and eliminating extreme poverty have been the target for Millennium development goals (MDG) achievement and during that period intensive implementation activities were carried out [2]. There is also a policy under implementation that states, increasing the use of contraceptives from 42% in 2015 to 55% in 2020. However, after many efforts, there were no adequate achievements related to the plan and there were no deserved changes [3]. Evidence clearly indicated that contraception prevents up to one-third of maternal losses. By agreeing with women to suspend motherhood, birth spacing, avoiding unplanned pregnancies, and avoiding unsafe abortions, and halting child bear when desired family size attained were very vital evidences to keep women and AGYW safe [4, 5]. The period from adolescence to young adulthood is crucial for physical, mental, and behavioral development. To have a healthy sexual life and successful reproductive period, AGYW face numerous challenges of accessing the resources they want modern contraceptive methods. Contraceptive methods use provide AGYW with a chance to make informed decisions about when and where to have children [6, 7].

In Sub–Sahara African countries, the number of pregnancies among adolescents and young women (AGYW) remained very high which is the sign of the low contraception use [8]. From the recent EDHS, the number of AGYW pregnancies was increased in the country [9]. In other study among the women who want to sidestep getting pregnant, the unmet need is disproportionately very high [10].

Whether they are married or not, the use of modern contraceptive among AGYW had very low compared to other age groups in the developing world. This means, the contraceptive need of the AGYW deserve further international interventions [10]. In other words, despite the introduction of modern contraception over the last some decades, the level of utilization was also not adequate among all reproductive age women. In Ethiopia only 36% of married women in reproductive age (15–49 years) used modern contraceptives [9]. Considering this understanding, the aspects of the influences of the modern contraceptive use among AGYW is very vital for designing interventions, plans, and policies to address premature age pregnancies and other related issues. It is also helpful to reduce unsafe abortions, maternal mortality, and sexually transmitted infections. There are several factors contributing for the low use of contraceptives amongst AGYW. Evidence have shown that there were some individual and community-level factors influencing contraceptive use [11–14]. Indeed, there was limited evidence regarding modern contraceptive use among AGYW in the country in the large samples and it has been difficult to follow the magnitude of the problem throughout the country. The areas which needed special attention were not appropriately mapped and that made the implementations fragmented. Studies which cover adequate proportion of the country’s area has been appealing to lead the interventions to avoid the fragmentation and to get focus. Therefore, the current study examined the individual and community-level predictors of contraceptive uses in Ethiopian among AGYW based on the data from the EDHS 2016. The findings of this study might be helpful and the information generate might be comprehensive evidence for planners, policymakers, program managers, researchers, and for all other stakeholders to improve the health of AGYW in Ethiopia.

## Methods

### Data source

A cross-sectional survey data from EDHS 2016 were used for this study. EDHS 2016 is the fourth nationally representative survey conducted in Ethiopia. The EDHS 2016 data was collected using a two-level multistage stratified cluster sampling to pick eligible respondents from rural and urban areas of Ethiopia. Different questionnaires were employed to collect data from women, men, couples, and children. The survey was intended to collect and deliver data on several demographic indicators, including sexual and reproductive health data like marriage, pregnancy, fertility, family planning, sexual behavior, maternal health, STIs, and HIV/AIDS [9]. In the current analysis, we included only AGYW (15–24 years) who were sexually active and were not pregnant during the survey from the dataset. The EDHS data were collected from participant by direct face-to-face interviews.

### Measurement of variables

#### Dependent variable

The dependent variable for the study is the current use of modern contraception. WHO defines ‘Adolescents’ as an individuals in the age group of 10–19 years and ‘Youth’ in the age group of 15–24-year [15]. We derived the dependent variable from the question that the women asked about the type of contraceptive methods she is using at the time of the survey. We then coded responses as “no method”, “folkloric method”, “traditional method” and “modern method”. Modern methods include male and female sterilization, injectables, intrauterine devices (IUDs), contraceptive pills, implants, female and male condoms, and emergency contraceptive methods. Periodic abstinence (rhythm, calendar method), withdrawal (coitus interruptus), lactational amenorrhea, and we labeled country-specific traditional methods. Locally and spiritually defined methods of unverified effective methods, such as herbs, amulets, and gris-gris methods were the folkloric methods. The existing EDHS data has already excluded women who were pregnant, and those who never had sex from the variables lists. For this study, we coded adolescents and young women using modern contraception methods as ‘1 = yes’ and recoded those not using any modern methods, those using traditional methods, and those using folkloric methods as ‘0 = no’.

#### Independent variables

##### Individual-level predictors

AGYW’s age at birth was obtained after subtracting the date of birth of AGYW in century month code (CMC) from date of birth of child in CMC. It was then grouped into 15–19 years, 20–24 years. AGYW’s educational status was categorized in to no education, primary, secondary, and technical/vocational or higher. Given the few respondents in vocational and higher categories, it was re-categorized in to: no education, primary, and secondary and above. Religion was categorized into the dominant religion groups as Protestant, Orthodox, Muslim and others. Marital status was defined as single, married, Widowed and divorced. Current working status (AGYW occupation status) was captured by AGYW who are currently have work or who have worked in the last 12 months and recorded as not working and other categories. Since there were other several working categories, it was re-categorized as not working and working. In EDHS, household wealth index was categorized in quintiles as: poorest, poor, average, rich and richest and for this category, principal component analysis was used. Then, we re-categorized the scale in to poor, middle, and rich for easy understanding. Age at first sexual intercourse was a continuous variable, but categorized into < 20 and ≥ 20. Parity is the number of viable children a woman might have. It was grouped in to no birth, one birth, two birth, and three and above births. Husbands’ desire for children was the plan of number of children by husbands. It was defined as husband want some, husband want more, husband want fewer, and don’t know. Abortion is any type of pregnancy ended before 28th weeks of gestation. It includes any spontaneous and non-spontaneous abortion performed for treatment or other purposes. Media exposure was described as hearing information from radio, TV, and newspaper.

EDHS assessed exposure to media by asking “Do you listen to the radio or watch to television (TV) at least once a week, less than once a week or not at all?”. These variables first categorized into “yes”, “no”, and not at all Exposure to media variable was considered “yes” if the subject was exposed to one or two of the medias, and said “no” otherwise. The summary of definition of some of the variables were provided in Table 1.

**Table 1:** The summary of definition of some of the variables

Variables	Definition/categories
Age of the AGYW	Obtained by subtracting date of birth of AGYW from century month code (CMC). It then recoded to 15–19 and 20–24 years
AGYW’s educational status	no education, primary, secondary, and technical/vocational or higher
Religion	Protestant, Orthodox, Muslim and others
Marital status	single, married, Widowed and divorced
Working/occupational status	Working currently, not working currently
House wealth index	poor, middle, and rich
Age at first sexual intercourse	Treated as a continues variable re-categorized in to < 20 and ≥ 20
Parity	no birth, one birth, two birth, and three and above births
Husband desire of children	husband want same, husband want more, husband want fewer, and don’t know
Health facility visits	Yes/no
Exposure to media	Yes/no

#### Community-level predictors

##### Region

There are nine regions (Tigray, Afar, Amhara, Oromia, Somali, Benishangul, SNNPR (south nation nationalities people’s region), Gambela, and Harari) and two city administrations (Addis Ababa and Dire Dawa) in the country. AGYW were selected from any of the eleven areas.

##### Residence

indicates whether an individual live in rural or urban or whether place is rural or urban.

### Data management and analysis

Descriptive statistics were applied to summarize the study variables as mean, standard deviation, percent or proportions. Before applying descriptive statistics weighting, technique was applied to account for disproportionate sampling and other segregations implemented during sampling. Bivariate analysis was conducted to identify variable that merit to be included in the model. Due to the sampling methods DHS apply, the dependence of responses from different levels of hierarchy was suspected. This implies that a single-level traditional statistical model might not be adequate to control for the clustering effect. Thus, since the data has individual and community levels, we applied multilevel binary logistic regression. The decision was made based on the Intra-Class Correlation (ICC), which showed high dependency due to the clustering of the data at the community level. Four consecutive models were built to identify predictors of modern contraceptives use. Model 1 is an empty (the intercept only model) employed before adding predictors [16]. model 2(fixed effect model) included all individual-level variables that were initially significant at *p*-value of < 0.25 to determine the level of variance explained by the model. Model 3 (random effect model) included cluster-level (community -level) variables and model 4(the mixed effect model) was the final model in which both the individual and community level variables introduced. All analyses were performed in STATA 14.2 and the output was presented using adjusted odds ratio (AOR) and 95% CI.

To determine the community effect, Intra-community Correlation (ICC) was estimated by applying the community level and individual level variances. Likelihood Ratio (LR) test, Median Odds Ratio (MOR), and Proportional Change in Variance (PCV) were also examined to check the fitness of the model using the following statistical formula.

ICC= $\frac{{\upsigma^2}_a}{{\upsigma^2}_a+{\upsigma^2}_b}$; where, σ^2^_*a*_ is the community level variance and σ^2^_*b*_ indicates individual level variance. The individual variance (σ^2^_*b*_) equal to π^2^/3 that is the fixed value.

MOR= *e* ^0.95*^ $\sqrt{V{a}_{\_1}}$, where, *Va*__1_ is the variance in the empty model.

PVC = $\frac{Va\_1- Va\_2}{Va\_1}$, where, *Va* _ 1 is variance of the empty model and *Va* _ 2 is neighborhood variance in the subsequent model).

### Ethical consideration

Data for this study was accessed from the Demographic Health Survey (DHS) website (http://www.dhsprogram.com). The procedure was confidential and we avoided any ways exposing households or individuals. To collect the data, EDHS obtained permission from the Ethiopian Health Nutrition and Research Institute (EHNRI) Review Board and the National Research Ethics Review Committee (NRERC) from the Ministry of Science and Technology. During the data collection, verbal informed consents were collected from participants and data collectors explained the purpose of the study for participants as published in 2016 EDHS report.

## Results

### Prevalence of modern contraceptive use among AGYW in Ethiopia

**Fig. 1:**
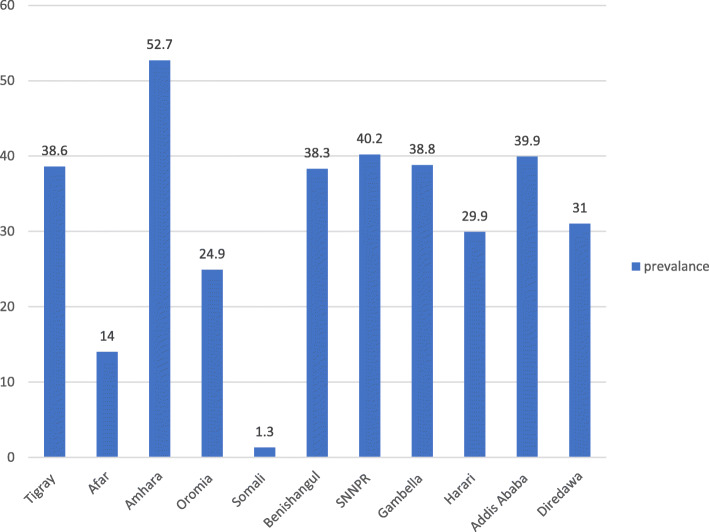
Prevalence of modern contraceptive use among women age (15–24) across region in Ethiopia 2016 EDHS

**Fig. 2:**
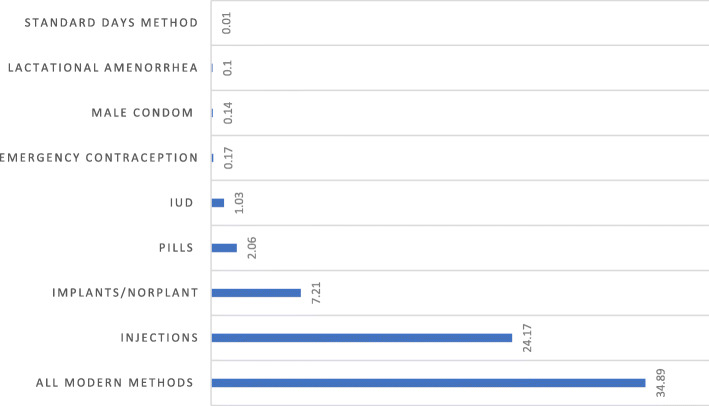
prevalence of modern contraceptive use by methods among adolescent and young women in Ethiopia

The prevalence of modern contraceptive use among AGYW in Ethiopia was 34.89% [95% CI, 0.32,0.36] in 2016. Across the regions, it was the highest in the Amhara region and lowest in Somali (Fig. 1). Modern contraception uses by method indicated, 24% injectable, 7.21% Implants/Norplant, and 2.06% used pills (Fig. 2).

A total of 2273 participants were extracted from the EDHS 2016 dataset. The age range of the participants was 15–24 within 5 years preceding the survey. Of these, 908 (39.90%) were from Oromia region and 7 (0.3) were from Harari region. About 1820(80%) of AGYW were live in rural residents, and more than two-third (72.7%) of them were aged 20–24 years. Half of the participants (51.2%) learned only primary education. Other descriptive statistics showed, 83.8% married, 42.6% Orthodox, 54.6% not-exposed to media, and 86.6% of the AYW had their first sexual intercourse before the age of 20. Over two-third AGYW (72.3%) had no work currently. Based on the wealth index category, only 41.9% of participants haven fallen the in rich index. The results further showed that 39.5% AGYW had no viable children, 43.9% were not given birth, and 4.9% of AGYW had at least one abortion (Table 2).

**Table 2:** Distribution of modern contraceptive use across individual and community-level factors among AGYW in Ethiopia (Weighted sample *N* = 2273)

Variables	Contraceptive use and weighted frequency		
	No (%)	Yes (%)	Weighted %
**Region**			
Tigray	123 (61.5)	77 (38.5)	200 (8.8)
Afar	27 (86)	4 (14)	31 (1.4)
Amhara	242 (47.2)	270 (52.8)	512 (22.5)
Oromia	682 (75.1)	226 (24.9)	908 (39.9)
Somali	81 (98.8)	1 (1.2)	82 (3.6)
Benishangul	18 (62.1)	11 (37.9)	29 (1.3)
SNNPR	227 (61.1)	152 (38.9)	379 (16.7)
Gambella	5 (55.6)	4 (44.4)	9 (0.4)
Harari	5 (71.1)	2 (28.9)	7 (0.3)
Addis Ababa	62 (61.2)	41 (39.8)	103 (4.5)
Diredawa	10 (67.7)	5 (33.3)	15 (0.6)
**Residence**			
Rural	1239 (68.1)	581 (31.9)	1820 (80)
Urban	241 (3.1)	213 (46.9)	454 (20)
**Age**			
15–19	427 (8.9)	193 (31.1)	620 (27.3)
20–24	1053 (63.7)	601 (36.3)	1654 (72.7)
**Respondent working**			
Yes	364 (57.7)	267 (42.3)	631 (27.7)
No	1116 (67.9)	527 (32.1)	1643 (72.3)
**Religion**			
Orthodox	511 (52.8)	457 (47.2)	968 (42.6)
Muslim	694 (82.)	146 (17.4)	840 (36.9)
Protestant	266 (9.)	180 (40.4)	446 (19.6)
Others	10 (50)	10 (50)	20 (0.9)
**Marital status**			
Single	149 (77.2)	44 (22.8)	193 (8.5)
Married	1187 (62.3)	719 (37.7)	1906 (83.8)
Widowed	11 (84.6)	2 (15.4)	13 (0. 6)
Divorced	133 (82.1)	29 (17.9)	162 (7.1)
**Educational status**			
No-education	472 (75.5)	153 (24.5)	625 (27.5)
Primary	749 (64.3)	415 (35.7)	1164 (51.2)
Secondary & higher	259 (46.5)	225 (53.5)	484 (22.3)
**Wealth status**			
Poor	693 (76.5)	213 (23.5)	905 (39.8)
Middle	264 (63.3)	153 (36.7)	417 (18.3)
Rich	524 (55.1)	428 (44.9)	952 (41.9)
**Parity**			
No-birth	620 (74.7)	277 (25.3)	897 (39.5)
One birth	577 (57.8)	422 (42.2)	999 (43.9)
Two birth	240 (73.8)	85 (26.2)	325 (14.3)
Three or more	44 (81.5)	10 (18.5)	54 (2.3)
**Abortion**			
No	1394 (0.4.4)	769 (35.6)	2163 (95.1)
Yes	86 (78.2)	24 (21.8)	110 (4.9)

### Factors associated with modern contraceptive

In this study, we found substantial association of individual and community-level factors with modern contraceptive use. We checked ICC, LLR tests, and deviance in the multilevel models. Deviance was used to compare the fitted models and model 3 with the lowest deviance value was the best-fitted model. In the null model, the ICC indicated that 32.7% of the total variability for modern contraceptive was due to differences between clusters while the remaining unexplained 67.3% of the total variability of modern contraceptive was attributable to the individual differences.

Therefore, it was merit to use multilevel logistic regression model to get an unbiased standard error. We used deviance for model comparison where the last model was the best-fitted model (model 3) (Table 3). In the multivariable multilevel analysis model, residence, region, religion, marital status, wealth status, working status, abortion/ever terminate a pregnancy, husband desire for children, parity, and TV exposure were significantly correlated with modern contraception use. The odds of using modern contraception among AGYW living in urban areas were 1.61 times [AOR = 1.61, 95% CI = 1.16, 2.45] higher than that of those living in rural areas. AGYW who belongs to Amhara [AOR = 5.02, 95% CI = 2.65, 8.30], SNNPR [AOR = 1.85, 95% CI = 1.05, 3.40], and Benishangul [AOR = 1.91, 95% CI = 1.0, 3.20] had higher odds of using modern contraceptive methods; however, Somali [AOR = 0.098, 95% CI = 0.04, 0.43] had lower odds of using modern contraception compared to Addis Ababa (Table 3).

**Table 3:** Multivariable multilevel logistic regression analysis results of both individual-level and community-level factors associated with modern contraceptive in Ethiopia, EDHS 2016

Individual-level and community-level characteristics	Model 0	Model 1	Model 2	Model 3
**Region**				
Addis Ababa	**–**	**–**	1	**1**
Tigray	**–**	**–**	2.08 [1.19,3.61]	1.44 [0.83,2.47]
Afar	**–**	**–**	0.33 [0.17,0.63] *	0.66 [0.38,1.46]
Amhara		**–**	4.94 [2.77, 8.81] **	5.02 [2.65,8.30] **
Oromia	**–**	**–**	1.35 [0.76,2.4]	1.24 [0.63,1.93]
Somali	**–**	**–**	0.044 [0.014,0.13] **	0.098 [0.04,0.43] **
Benishangul	**–**	**–**	2.05[1.12,3.76]	1.91 [1.0,3.20] *
SNNPR	**–**	**–**	2.76 [1.54,4.93] *	1.85 [1.05,3.40] *
Gambella	**–**	**–**	1.25 [0.70,2.24]	1.20 [0.69,2.19]
Harari	**–**	**–**	1.04[0.58,1.90]	1.03 [0.59,1.93]
Diredawa	**–**	**–**	0.89 [0.50,1.59]	1.25 [0.77,2.41]
**Residence**				
Rural	**–**	**–**	**1**	**1**
Urban	**–**	**–**	2.94 [2.15,4.02] **	1.61 [1.16,2.45] **
**Age**				
15–19	**–**	1	**–**	**1**
20–24	**–**	1.02 [0.78,1.3]	**–**	0.95 [0.74,1.28]
**Respondent working**				
No	**–**	**1**	**–**	**1**
Yes	**–**	1.34 [1.06,1.69] *	**–**	1.36 [1.06,1.71] *
**Religion**				
Muslim	**–**	**1**	**–**	**1**
Orthodox	**–**	4.77 [2.8,5.0] **	**–**	2.77 [2.00,3.38] **
Protestant	**–**	2.75 [1.6,3.1] **	**–**	1.94 [1.30, 2.89] **
Others	**–**	2.29 [0.59,8.81]		1.92 [0.51,7.21]
**Marital status**				
Single	**–**	1	**–**	**1**
Married	**–**	2.33 [1.56,3.5] **	**–**	2.01 [1.33,3.04] **
Widowed	**–**	0.67 [0.07,7.4]	**–**	0.99 [0.08,11.96]
Divorced	**–**	0.86 [0.49,1.52]	**–**	0.72 [0.41,1.30]
**Educational status**				
Secondary & higher	**–**	1	**–**	**1**
Primary	**–**	1.24 [0.95,1.63]	**–**	1.19 [0.91,1.56]
No-education	**–**	0.72 [0.55,1.15]	**–**	0.80 [0.58,1.24]
**Wealth status**				
Poor	**–**	1	**–**	
Middle	**–**	2.13 [1.5,3.0] **	**–**	1.90 [1.32,2.65] **
Rich	**–**	2.17 [1.6,2.94] **	**–**	1.99 [1.35,2.68] **
**Age at first sex**				
< 20 year	**–**	1	**–**	**1**
20–24 year	**–**	1.02 [0.73,1.39]		1.11 [0.79,1.51]
**HF visited last 12 month**				
No	**–**	1	**–**	**1**
Yes	**–**	1.2 [0.95,1.47]		1.18 [0.92,1.45]
**Parity**				
No-birth	**–**	1	**–**	**1**
One birth	**–**	2.56 [1.95,3.37] **	**–**	2.66 [2.06,3.60] **
Two birth	**–**	1.66 [1.12,2.48] *	**–**	1.84 [1.28,2.90] *
Three or more	**–**	1.57 [0.73,4.10]	**–**	2.47 [1.06,6.35] *
**Abortion**				
No	**–**	1	**–**	**1**
Yes	**–**	0.44 [0.26,0.77] *	**–**	0.47 [0.26,0.80] *
**Husband desire for children**			**–**	
Both want same	**–**	1	**–**	**1**
Husband want more	**–**	0.56 [0.39,0.7] **	**–**	0.59 [0.41,0.78] *
Husband want fewer	**–**	0.75 [0.421.35]	**–**	0.73 [0.40,1.30]
Don’t know	**–**	0.55 [0.4,0.73] **	**–**	0.50 [0.39,0.68] **
**Radio exposure**				
No	**–**	1	**–**	**1**
Yes	**–**	1.08 [0.84,1.39]	**–**	1.07[0.84,1.38]
**Newspaper exposure**				
No	**–**	1	**–**	**1**
Yes	**–**	1.0 [0.73,1.36]	**–**	1.04[0.77,1.41]
**TV exposure**				
No	**–**	1	**–**	**1**
Yes	**–**	1.62 [1.21,2.16] *	**–**	1.6[1.17,2.20] *
**Model comparison and random effect**				
ICC	0.327 [0.26,0.39]	–	**–**	–
Log-likelihood	− 1441.8	− 1205	− 1319.64	− 1162.5
Deviance	2883.6	2410	2639.28	2325

*Key: 1: reference group; []: confidence interval, *p*-value 0.05–0.01 *: *p*-value < 0.01 **

At the individual level, there were eight variables significantly correlated to modern contraception use. The odds of modern contraception use among AGYW was higher among Orthodox Christian [AOR = 2.77, 95% CI = 1.77, 3.28] and Protestant religions [AOR = 1.94, 95% CI = 1.14, 2.53]. Married AGYW had higher odds of using modern contraception [AOR = 2.01, 95% CI = 1.39, 3.16] relative to single AGYW. Those who were in the middle [AOR = 1.90, 95% CI = 1.32, 2.65] and rich [AOR = 1.99, 95% CI = 1.35, 2.68] wealth index had higher odds of using modern contraception relative to the poor wealth index. Currently working AGYW had 1.36 times higher odds of using modern contraception compared to not working AGYW [AOR = 1.36, 95% CI = 1.06, 1.71]. Those AGYW who had at least one viable birth were more likely correlated with modern contraception use [AOR = 2.66, 95% CI = 2.06, 3.6], while having two viable births [AOR = 1.8495% CI = 1.28, 2.90] and three or more births [AOR = 2.47, 95% CI = 1.06, 6.35] were more likely associated with the modern contraception use compared to no-birth. Husband’s desire for children had 41% reduced contraceptive use [AOR = 0.59, 95% CI = 0.41, 0.78] and those husbands who don’t know their desired number of children had 50% reduced modern contraceptive use [AOR = 0.50, 95% CI = 0.39, 0.8]. AGYW who had abortion had 53% reduced contraception use [AOR = 0.47, 95% CI = 0.26, 0.80] than AGYW who never had abortion. Furthermore, AGYW having media exposure had 1.6 times [AOR = 1.6, 95% CI = 1.17, 2.20] higher odds of using contraceptive compared to the counterparts (Table 3).

## Discussion

In the current study, we investigated the individual and community-level predictors correlation with modern contraceptive use among AGYW in Ethiopia. The prevalence of modern contraception uses among 2273 women aged (15–24 years) was 34.9%. The finding was consistent with the finding in Republic of Congo 31.7% [12] and lower compared to the findings in other low and middle-income countries such as 56% in Malawi, 55% in Lesotho, 50% in Tunisia [17], 58% in South Africa [18], and 57.5% in Kenya [19].

At individual-level, married AGYW, middle and above wealth index, Orthodox and protestant religion, having more than one viable child, husband desire of children, ever abortion, urban place of residence, and TV media exposure were positively associated with modern contraceptive uses while current status of not working and living Somali regions were negatively correlated with modern contraceptive use.

AGYW who relayed on modern contraceptive methods were five-fold in Amhara, and nearly 2 folds in SNNPR and Benishangul; however, it was 90.2% decreased in Somali regions compared to Addis Ababa. This might be related to the variations in the availability of resources and accessibility of different family planning-related services across these administrative regions; while awareness might also be a big factor for variation. It is consistent with the results of previous studies in Ghana [20] and Nigeria [21]. In the current study, AGYW from urban settlements used contraception nearly 2 folds than those from rural settlements. The reason might be still the availability, accessibility of the resources, and awareness. This finding is supported by studies done in Uganda [22], Nigeria [23], and Afghanistan [24]. However, this finding was inconsistent with the study in Ghana and Indonesia where rural resident women were the most users of modern contraceptive methods [25, 26]. The reason was not clear and might be due to the intention to discover but also could be due to poor knowledge of cultural restrictions on contraception in some areas.

Orthodox and Protestant religion followers were found to be the better users of modern contraceptive methods in Ethiopia. The finding is consistent with that of the study done in Zambia [27], Ethiopia [28, 29], and Nigeria [30]. The reason might be due to the fact that religion might have similar socio-cultural importance in influencing the life of women in these countries in the way. Especially, the introduction of some family planning teachings in religiously non-conservative countries might have uttermost advantageous.

AGYW in the middle and richer wealth index quintiles were more likely to use modern contraceptive methods relative to the poor wealth category. A similar finding is recognized in studies conducted in different African countries like Nigeria [23], Tanzania [31], and Ghana [32]. Poor women might face difficulties in getting access to services and financial control might be the source of the limitation to exercise one’s rights. Our study also revealed that married AGYW had a better tendency to use modern contraceptive methods. The result was comparable with the studies in Ethiopia [33], Ghana [34], and in conflict with the study done in Mali [35]. The possible explanation might be the exposure to maternal and birth control services during antennal and postnatal cares. The exposure might probably increase mothers’ access to scarce resources and enabled them to use it. The result could also indicate the importance of couple motivation through education, self-reliance of married women, and male participation in reproductive health issues.

Employment was directly associated with the use of contraception. This provides prominent evidence that women’s employment could raise the use of modern contraception in Ethiopia and other studies also confirmed the same findings [36, 37]. This might also be due to the increase in self-reliance, self-sufficiency, less dependency to make self-decisions, affording service, and access.

The use of modern contraception was directly associated with births and parity. As the number of births increased, the use of modern contraceptive methods was also increased which is consistent with the previous studies in some African countries [38, 39]. This clearly showed the improvement in the behavioral change of the women that reflected by the intention of reducing the number of births as they stay long in marriage. This might take women to use contraception methods; however, the husband’s desire for children affected contraceptive use of AGYW. Similar studies in Ethiopia [40] and Indonesia [26] revealed consistent outcomes. This indicates, women empowerment might be another area for intervention to increase contraception use. AGYW women who ever had abortion were not using contraception compared to those who did not have abortion. This is consistent with the study in Zambia [27] and might indicates that those who had no chance of accessing, affording, economically incapable of supporting themselves are always at risk of such pregnancy outcomes and might not have a chance to overrun the problem. Exposure to media had shown vital in our study, as it directly affected modern contraceptive use. This finding is consistent with the studies conducted in Bangladesh [41] and South Asia countries [42]. The mass media is a good method to reach millions of AGYW so that countries need to increase the effort more than ever as the problem persists. Although this study found crucial points like how the modern contraceptive use followed religious and dwindled in conservative region, how visual media exposure had positive influence on contraceptive method uses, and all other marvel findings, it also has some limitations that need to be considered while applying the findings of this study.

Disproportionate sample, using third party data, and cross-sectional nature of the study was some of the limitations. To handle disproportionate sampling, we applied weighting to the variables before analysis. To deal with third party nature of the data, we applied all the necessary protocols which are internationally standards to each country and described all methods and manipulation of the data under method section. We also declare to readers that the magnitude of the variables like wealth index, educational status, and working status might be different today as we analyzed data from EDHS 2016.

## Conclusion

In the last 15 years, modern contraceptive use among AGYW in Ethiopia has enhanced; however, the prevalence is still little equated to the prevalence in other African countries and global indicators. At individual-level marital status, wealth index, religion, working status, parity, husband desire children, abortion, and TV exposure were significant predictors for modern contraception. Region and resident were found as a community-level factor associated with modern contraceptive use among AGYW. Therefore, policymakers, governmental and non-governmental organizations could strength the efforts towards family planning health services particularly for rural residents and might design effective public health interventions in identified regions. Predictors of Modern contraceptive use are not only individual characteristics of AGYW but also community-level factors that determine it. Thus, efforts to rise modern contraceptive use in Ethiopia must emphasize both at individuals and community-level hard works equally.

## Data Availability

The data in which the authors used to produce this manuscript are available upon reasonable request from http://www.dhsprogram.com.

## Abbreviations

AOR: Adjusted Odds Ratio,
CI: Confidence Interval
EDHS: Ethiopia Demographic and Health Survey
ICC: Intra Class Correlation Coefficient
LLR: Log-Likelihood Ratio
MOR: Median odds ratio
OR: Odds Ratio
PVC: Proportional Change in Variance
SNNPR: Southern Nations, Nationalities, and Peoples’ Region
